# Dichloroacetate as a possible treatment for endometriosis-associated pain: a single-arm open-label exploratory clinical trial (EPiC)

**DOI:** 10.1186/s40814-021-00797-0

**Published:** 2021-03-12

**Authors:** H. W. Leow, M. Koscielniak, L. Williams, P. T. K. Saunders, J. Daniels, A. M. Doust, M-C Jones, G. D. Ferguson, Y. Bagger, A. W. Horne, L. H. R. Whitaker

**Affiliations:** 1 MRC Centre for Reproductive Health, Queen’s Medical Research Institute, University of Edinburgh, 47 Little France Crescent, Edinburgh, EH16 4TJ UK; 2 Usher Institute, NINE Edinburgh BioQuarter, 9 Little France Road, Edinburgh, EH16 4UX UK; 3 Centre for Inflammation Research, Queen’s Medical Research Institue, University of Edinburgh, Edinburgh, EH16 4TJ UK; 4 Clinical Trials Unit, University of Nottingham, University Park, Nottingham, NG7 2RD UK; 5 Institute of Clinical Sciences, University of Birmingham, Edgbaston, Birmingham, B15 2TT UK; 6 Reproductive Medicine and Maternal Health, Ferring Research Institute, San Diego, CA 92121 USA

**Keywords:** Chronic pelvic pain, Gynaecology, Glycolysis, Repurposing, Feasibility trial

## Abstract

**Background:**

Endometriosis (where endometrial-like tissue is found outside the uterus) affects ~ 176 million women worldwide and can lead to debilitating pelvic pain. There is an unmet need for new medical treatment options for endometriosis. Pelvic peritoneal mesothelial cells of women with endometriosis exhibit detrimental metabolic reprogramming that creates an environment favouring the formation and survival of endometriosis lesions. We have generated powerful preclinical proof-of-concept data to show that it is possible to correct this metabolic phenotype using dichloroacetate (DCA), a non-hormonal compound previously used to treat rare metabolic disorders in children. We plan a single-arm, open-label, single site exploratory clinical trial to inform the design of a future randomised controlled trial (RCT) to determine the efficacy of DCA for the treatment of endometriosis-associated pain.

**Methods:**

We will recruit 30 women with endometriosis-associated pain over a 6-month period. All participants will receive approximately 6.25 mg/kg oral DCA capsules twice daily for 6 weeks, with a dose increase to approximately 12.5 mg/kg twice daily for a further 6 weeks if their pain has not been adequately controlled on this dose regime and side-effects are acceptable. If pain is adequately controlled with minimal side-effects, the lower dose will be continued for a further 6 weeks. The primary objective is to determine whether it is possible to achieve acceptable recruitment and retention rates within the defined exclusion and inclusion criteria. Secondary objectives are to determine the acceptability of the trial to participants, including the proposed methods of recruitment, treatment, follow-up frequency and number of questionnaires. The recruitment rate will be determined by the proportion of patients recruited from the pool of eligible women. The retention rate will be determined by the proportion of participants who attended the final trial visit.

**Discussion:**

This is a feasibility study to explore effectiveness and acceptability of the proposed field methodology (recruitment, retention, study processes and compliance with treatment). The results will be used to inform the design of a future RCT.

**Trial registration:**

ClinicalTrials.gov, NCT04046081 Registered 6 August 2019

## Background

Endometriosis is characterised by the presence of endometrial-like tissue outside the uterus, commonly on the lining of the pelvic cavity [1]. It is a chronic, inflammatory gynaecological condition that affects approximately 10% of women of reproductive age [2]. It is associated with debilitating pelvic pain, pain during menstruation, pain during intercourse and subfertility [3, 4]. Direct healthcare costs amount to nearly 3000 Euros per woman per year (based on 2009 prices), similar to those of diabetes mellitus [5]. Current treatment options are often unsatisfactory. Of women undergoing surgery, over half would have a further surgical procedure by 5 years [6]. Available hormonal treatments have unwanted side-effects and are contraceptive [7]. There is an ‘unmet need for new medical treatments for endometriosis’ which are disease modifying and fertility sparing [8].

Endometriosis exhibits cancer-like features [9]. Specifically, tumour cells are programmed by TGF-β1 to use aerobic glycolysis resulting in increased secretion of lactate [10–12]. We have shown that TGF-β1 and lactate are both elevated in the peritoneal fluid of women with endometriosis and that this is paralleled by a switch from normal mitochondrial respiration towards glycolysis in the human peritoneal mesothelial cells (HPMC) that line the pelvic cavity [10–12]. In the tumour microenvironment, lactate is considered a key factor in driving cell invasion, angiogenesis and immune suppression [13], all of which are implicated in the establishment and survival of endometriosis lesions. Using in vitro models, we have demonstrated that the glycolysis inhibitor, dichloroacetate (DCA), reversed the aberrantly increased glycolysis of HPMC and oral administration of DCA reduced the size of endometriosis lesions in a mouse model [14].

We plan a single-arm open-label exploratory clinical trial to determine the feasibility of achieving an acceptable recruitment and retention rates. This trial will inform the design of a potential future large randomised controlled trial (RCT) to determine the efficacy of DCA to treat women with endometriosis-associated pain.

## Methods/design

### Primary outcome

To determine whether it is possible to achieve acceptable recruitment and retention rates within the defined inclusion/exclusion criteria

### Secondary outcomes

- To determine the acceptability to participants of the proposed method of recruitment, treatment, questionnaires and follow-up (e.g. frequency and method for collecting pain scores and for completion of questionnaires)
- To assess how well-tolerated is DCA in women with endometriosis
- To determine if we can detect systemic DCA in blood samples of women with endometriosis
- To determine participants’ compliance with treatment and to assess the tools used to measure compliance

### Study design

This is a single site, open-label exploratory study of women with endometriosis.

### Study population

We will recruit 30 women with endometriosis-associated pain over a 6-month period. The duration of the entire trial will be 12 months. The trial visits will take place within NHS Lothian, UK. The participants will be required to come to the hospital for five visits, at day 1 and at weeks 2, 6, 8 and 12 to complete questionnaires, give blood samples and receive further DCA supplies if required. The participants will be taking the treatment for 12 weeks and will receive a phone call 4 weeks (± 1 week) after the last date of treatment to ask about their general health and how satisfied they are with the treatment and trial methods (see Table 1).

**Table 1:**
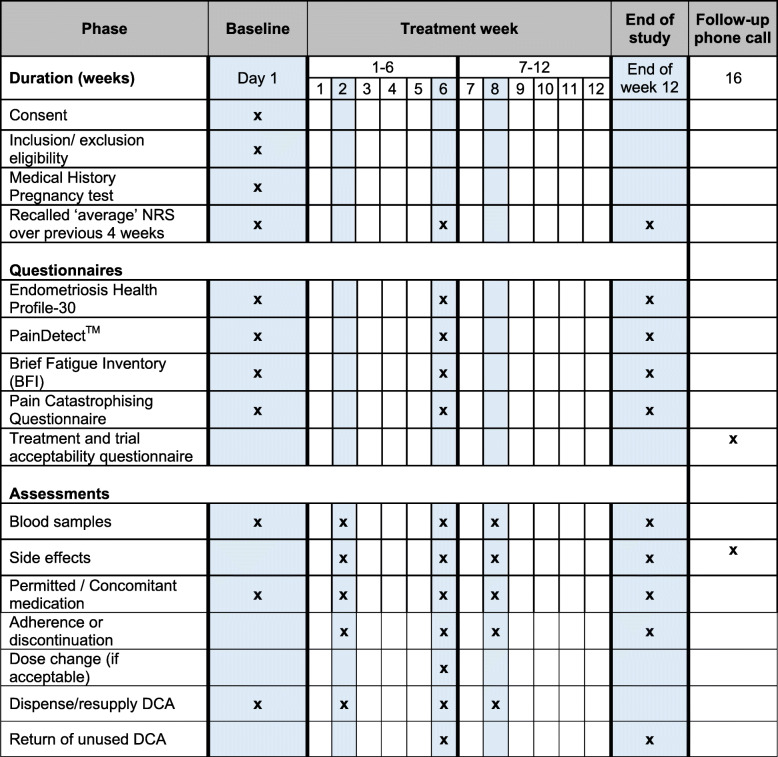
Summary of study visits and assessments

### Inclusion criteria

- Pre-menopausal women aged 18 or over
- Weight between 50 and 100 kg
- Superficial peritoneal endometriosis (ASRM stage I or II) identified at laparoscopy, performed within the last five years (and > 2 weeks from surgery)
- Pelvic pain for longer than 6 months
- Average pain score of ≥ 4 over the 4 weeks prior to treatment
- Willing to comply with the treatment
- Willing to use non-hormonal contraception throughout the trial
- Willing and able to complete informed consent

### Exclusion criteria

- Evidence of ovarian endometrioma or deep endometriosis (based upon current surgical staging or most recent imaging)
- Women who are pregnant or actively trying to get pregnant
- Known allergy or hypersensitivity to any excipient of DCA
- Breastfeeding
- Clinical evidence of pre-existing peripheral neuropathy
- Diabetes, history of liver, or history of kidney disease
- Taking part in another Clinical Trial of an Investigational Medicinal Product (CTIMP) or other interventional non-CTIMP studies
- Patient on combination anti-retroviral therapy
- History of malabsorption syndrome or substantial amount of small bowel or stomach removed

### Participant enrolment

Potentially eligible women will be asked by their attending clinician if they would be interested in participating in the study. If they agree, the women will then be given a patient information sheet and will be referred to members of the clinical research team to discuss the study in more detail. Patients will be allowed to self-refer to the study by contacting the research team directly. Informed consent will only be taken by a member of the research team once the participants have had ample time (at least 24 h) to read the patient information sheet and had their questions answered. Following consent, eligibility will be assessed by the clinical team. A screening log will be maintained and the following anonymised information will be monitored and collected for all potential participants: date of attendance in clinic, year of birth, reason for non-eligibility and reason for not participating if eligible and willing to give a reason. Ineligible and non-recruited participants will be offered routine NHS gynaecological care.

### Intervention

Based on our current preclinical studies and the current literature, we have chosen a dose which will potentially be effective for endometriosis-associated pain and has the least side-effects. Trials using DCA as a potential treatment for cancer or pulmonary arterial hypertension suggest that doses of 6.25 mg/kg BD should produce blood levels within the range required for pyruvate dehydrogenase kinase (PDK) inhibition, although DCA pharmacokinetics may be variable [15]. We also note that most side-effects, such as reversible peripheral neuropathy, seem to occur at doses of 25–50 mg/kg/day [16–18]. We therefore plan to trial the feasibility of a dose change with DCA at approximately 6.25 mg/kg BD (oral) for 6 weeks, followed by a dose increase to approximately 12.5 mg/kg BD (oral) for the next 6 weeks, depending on participant’s tolerance of treatment (total treatment phase of 12 weeks).

The drug will be provided in capsules containing 333 mg or 500 mg of DCA powder (Curaltus Ltd, Lithuania). Participants will be required to take between two to six capsules orally a day depending on the dose calculated using their body weight. The drug will be dispensed on day 1 of treatment and resupplied if needed at each visit during week 2, 6 and 8. Sufficient amount of drug will be provided to last until the next visit or end of study.

**Fig. 1:**
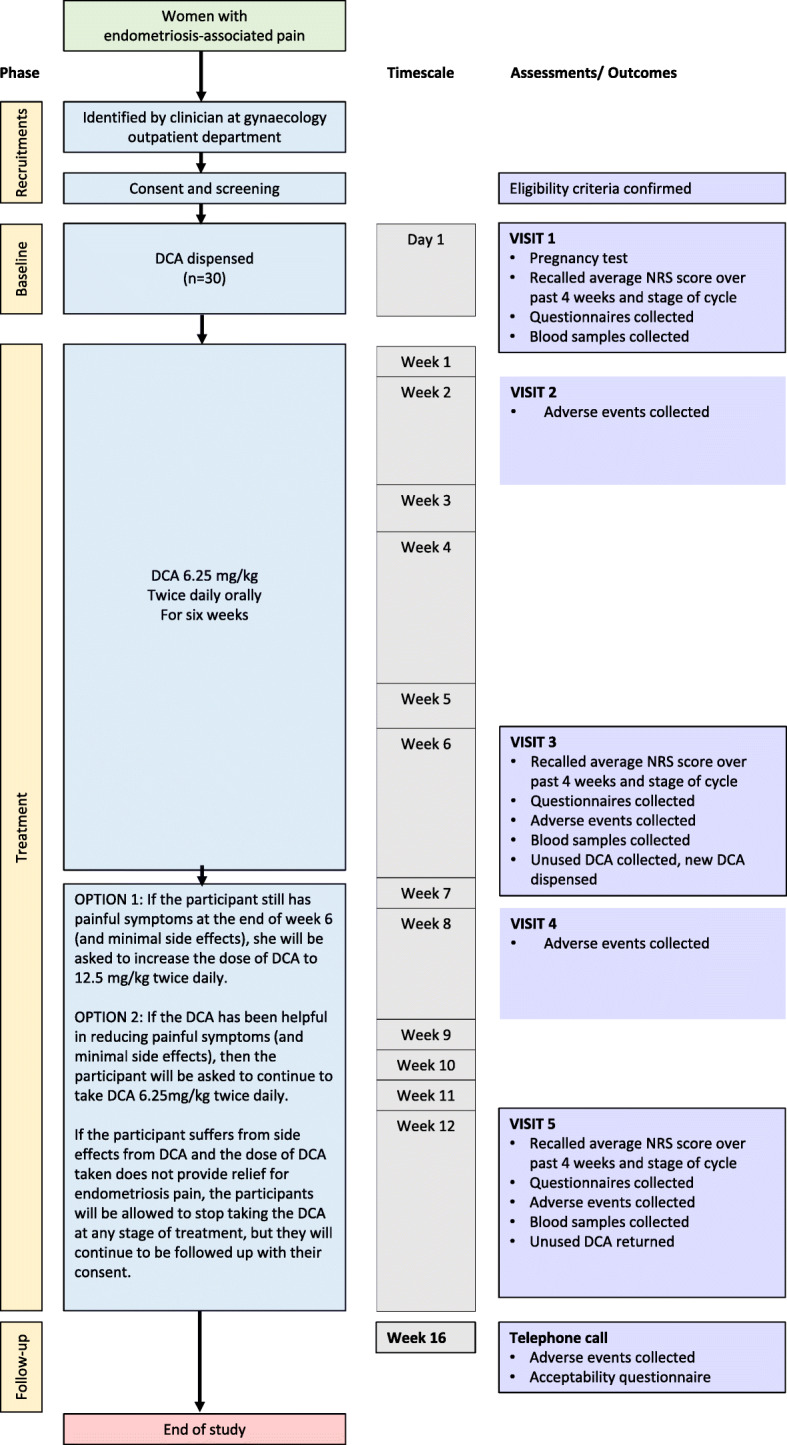
Study Flow chart. DCA, Dichloroacetate. NRS, Numerical Rating Scale

1. *Weeks 1–6*. All participants will receive approximately 6.25 mg/kg BD daily (oral) for 6 weeks.
2. *Weeks 6–12*. Dosing may be escalated depending on symptom control and side-effects (options 1–3 as below).
  - *Option 1*. If the participant still has painful symptoms at the end of week 6 and minimal or no side-effects, she will be asked to increase the dose of DCA to approximately 12.5 mg/kg BD daily (oral) for a further 6 weeks (see Figure 1, study flow chart). Dose escalation will be overseen by a medical doctor on the delegation log.
  - *Option 2*. If DCA has been helpful in reducing painful symptoms and caused minimal side-effects, then the participant will be asked to continue to take DCA 6.25 mg/kg BD daily (oral) for a further 6 weeks.
  - *Option 3*. If the participant suffers from side-effects from DCA and the dose of DCA taken does not provide relief for endometriosis pain, the participant is allowed to stop taking the drug at any stage of the treatment, but they will continue to be followed up with their consent.

If participants who take 12.5 mg/kg BD (oral) have side-effects and do not tolerate the treatment, they will be allowed to return to low dose of 6.25 mg/kg BD. If participants do not have enough capsules to return to low dose, they will be resupplied with new capsules.

### Assessment of participant compliance

Participants will be asked about compliance at each visit, which will be recorded in the case report form (CRF). Compliance will be also measured via a treatment diary and systemic blood levels.

### Other medications

Participants will be allowed to take any oral analgesics and alternative treatments (e.g. acupuncture) throughout the study period, which will be recorded in the CRF. Due to the potential for pain relief from hormonal therapy, patients will only be allowed to take hormonal therapies if they have started them more than three months before consent.

### Safety assessments

At the first visit, a medical history will be taken to assess specific eligibility points, such as concurrent neuropathy, diabetes, liver or kidney disease, anti-retroviral use and use of concomitant medications. Contraception requirement and method will be documented and a pregnancy test undertaken. Participants will be assessed at each visit by a Good Clinical Practice (GCP)-trained medical doctor to confirm their wellbeing, including a neurological examination to assess for peripheral neuropathy.

### Study assessments

Study visits and assessments are outlined in Table 1. Stage of cycle will be documented at commencement of treatment.

Quality of Life and pain questionnaires include the Endometriosis Health Profile-30 [19], PainDETECT^TM^ [20], Brief Fatigue Inventory (BFI) [21] and Pain Catastrophising Questionnaire [22]. At the end of the study (week 16) participants will be asked to complete an acceptability questionnaire, using a five point Likert scale. This consists of 11 questions to record acceptability of the proposed methods of recruitment, follow-up, treatments and use of quality of life and pain questionnaires. All questionnaires will be completed in private and anonymised. Patients will have a choice to complete them on paper during their visit or at home using an online link to secure REDCap database. Numerical Rating Scale (NRS) for pain will be collected on paper CRF only and will be recorded on the database that they were completed.

### Adverse events

At each visit, participants will be asked about any adverse events (AEs) that have occurred during treatment. Any AEs that occur after joining the trial will be reported in detail in the participant’s medical notes and AE log, and followed up until resolution of the event. All AEs will be assessed for seriousness, causality, expectedness and severity. All serious adverse events (SAEs) will be reported to the Academic and Clinical Central Office for Research and Development (ACCORD) Research Governance (http://www.accord.ed.ac.uk) and Quality Assurance Office based at the University of Edinburgh within 24 h of becoming aware that an SAE has occurred. Known side-effects of DCA (tingling or numbness, sleepiness, confusion, heartburn, mental fogginess and nausea) will not be recorded as AEs but will be recorded on the CRF at each visit. Admittance to hospital for surgery related endometriosis/pelvic pain, exacerbation of pain related to endometriosis/pelvic pain, or elective treatments planned prior to enrolment will not be reported as SAEs.

### Termination of study

All participants will be required to stop DCA treatment after 12 weeks and return any remaining DCA to the investigator at the end of the study. Participants may withdraw from the trial at any point or a participant can be withdrawn by the investigator. Although pregnancy is not considered an adverse event, any participant’s pregnancy will be recorded on a Pregnancy Notification Form and submitted to the ACCORD office within 14 days of being made aware of the pregnancy. All pregnant participants will be followed up until the outcome of the pregnancy. Data collection is envisaged to be completed in August 2020.

### Sample size

The emphasis in this study is to establish feasibility, not statistical significance. This study is designed primarily to explore the effectiveness of the proposed field methodology: recruitment, retention, study processes and compliance with treatment. We will aim to recruit as many women as possible over a 6-month period. We estimate that we will recruit 5–6 patients per month and will aim to recruit 30 patients. Data from this exploratory study will be used to refine sample size calculations for any future RCT.

### Statistical analysis

Summary data will be used to describe baseline characteristics: mean and standard deviation, or median and interquartile range (IQR), and minimum, maximum for continuous variables and number (percentage) of individuals for categorical variables. Since this is a single-arm trial with no randomisation, there is no risk of patients not receiving their randomised treatment. However, patients will be assumed to have completed the treatment as prescribed in the primary analysis, unless they failed to start treatment.

### Primary endpoint

The primary endpoint is to determine recruitment and retention rates. Using the information collected from the participant log, we will determine the number of patients recruited from the pool of eligible women and a 50% of 60, 95% CI (36.8–63.2%) recruitment will be deemed acceptable. While a retention rate of 100% would be ideal, we will consider a rate of 80% satisfactory. We will provide an estimate of the proportion and its 95% CI (61.4–92.3%). In addition, we will determine the nature and number of unanswered questions in each questionnaire. We aim to determine whether the trial design will perform well enough in the field to warrant rolling out the study to full trial. All analysis will be carried out in the University of Edinburgh.

### Secondary endpoints

Acceptability of proposed methods of recruitment, follow-up, treatments and questionnaires used in this study will be assessed quantitatively using self-reported side-effects and acceptability questionnaires at the end of study. We also aim to determine if treatment is acceptable in terms of self-reported compliance (from treatment diaries). The blood samples collected from patients will be analysed at University of Edinburgh, QMRI, to detect the systemic levels of DCA to confirm participants’ compliance. The assay will be developed in the research laboratory of the Edinburgh Clinical Research Facility. It will be analysed using mass spectrometry (choosing the instrument that will provide most accurate readings) and either Analyst MultiQuant software or Xcalibur Quantitate software. Descriptive statistics will be used to describe use of rescue analgesia, side-effects and compliance and will be compared by dose escalation or not.

### Data storage

All consented participants will be allocated a trial participant number. A REDCap database will be used to record the data, entered by the member of the research team. The data from the database will be used for analysis of the trial and will be archived for a minimum of 15 years. The online questionnaires completed by patients will be directly linked to the REDCap database.

## Discussion

Dichloroacetate (DCA) may be a potential novel non-hormonal treatment for endometriosis-associated pain in women. To date, this is the first study investigating DCA for endometriosis pain and assessing whether it is possible to achieve acceptable recruitment and retention rates. Given that many trials struggle with recruitment and often request extensions or become at risk of closure [23], investigating the feasibility and acceptability of treatments or interventions is of great importance to inform design of future definitive controlled trials [24]. If there are any indicators of problems with feasibility and acceptability affecting recruitment, retention, or safety, those relevant procedures will be revised and modified.

Our sample size of 30 women and 6 months recruitment period would be reasonable to provide a justified information of feasibility, acceptability to methodology (recruitment, retention, treatment, follow-up frequency), compliance rate and response to questionnaires. A key strength of the study is the clear definition of participant’s disease as endometriosis diagnosed via laparoscopy within the past five years (excluding deep endometriosis), and the specific inclusion and exclusion criteria would be reflective to the patient’s eligible to be on DCA in the future RCT based on their suitability for DCA as treatment option.

The questionnaires used to measure the physical and emotional functioning were selected based on the Initiative on Methods, Measurement, and Pain Assessment in Clinical Trials (IMMPACT) recommendations for chronic pain trials which would provide a reflective account on the women’s experiences [15–18]. Although pain scores and satisfaction rates are being used as part of the study protocol, the trial is not designed nor powered for formal analysis of effectiveness. The questionnaire results (e.g. pain score, physical and emotional functioning) from this study will not be carried forward for future studies. However, the nature and number of unanswered questions in the questionnaire can help us to assess suitability of the questionnaires and identify important outcome measures for future study.

This study is limited to examining the feasibility and acceptability of DCA intervention within a single tertiary centre setting and does not examine the feasibility of a multi-centre trial. This study does not assess the willingness of participants to be randomised and therefore cannot estimate the recruitment rate of future RCT but allow estimation of number of eligible patients and estimation of time frame for recruitment and collection of data in future RCT. The results may not be truly representative of all patient demographics due to selection bias as participants are recruitment from a single centre and must be willing to comply with the frequent follow-up visits.

DCA offers promise for a novel, non-hormonal medical therapy for the treatment of endometriosis-associated pain. The success of this study will depend on developing appropriate recruitment strategies. Ideally, we would want to meet both our recruitment and retention targets before consideration of a larger randomised controlled trial. However, Covid-19 social distancing guidelines and the closure of many outpatient gynaecology clinics during the lockdown is making the collection of follow-up data more difficult as patients are less willing to attend non-urgent hospital appointments. Thus, our estimates of both recruitment and retention are likely to be underestimates of these figures under normal circumstances. Nonetheless, this study will still give us an estimate of the lower limit of these figures, which can then be used to inform a future clinical trial comparing the effectiveness of DCA to placebo administration for endometriosis-associated pain.

## Data Availability

Data sharing is not applicable to this article as no datasets were generated or analysed during the current study.

## Abbreviations

ACCORD: The academic and clinical central office for research and development
AEs: Adverse events
ASRM: American Society of Reproductive Medicine
BFI: Brief fatigue inventory
CRF: Case report form
CTIMP: Clinical trial of an investigational medicinal product
DCA: Dichloroacetate
GCP: Good Clinical Practice
HPMC: Human peritoneal mesothelial cell
IMMPACT: Initiative on methods, measurement, and pain assessment in clinical trials
IQR: Interquartile range
NHS: National Health Service
NRS: Numerical rating scale
PDK: Pyruvate dehydrogenase kinase
RCT: Randomised controlled trial
SAEs: Serious adverse events
